# Association Among Systemic Immune-Inflammation Index, Vitamin D Status and Clinical Subtypes of ADHD: A Retrospective Investigation

**DOI:** 10.62641/aep.2224

**Published:** 2026-10-15

**Authors:** Tingting Lu, Yingying Chen, Feiping Hu, Yingping Zhu

**Affiliations:** ^1^Department of Child Health Care, Dongyang Maternal and Child Health Hospital, 322100 Jinhua, Zhejiang, China; ^2^Healthcare Department, Dongyang Maternal and Child Health Hospital, 322100 Jinhua, Zhejiang, China

**Keywords:** attention-deficit/hyperactivity disorder, systemic immune-inflammation index, 25-hydroxyvitamin D, neuroinflammation, paediatric ADHD

## Abstract

**Background:**

Attention-deficit/hyperactivity disorder (ADHD) has been linked to immune dysregulation and nutritional imbalance. The systemic immune-inflammation index (SII) is a composite inflammatory marker, while serum 25-hydroxyvitamin D (25(OH)D) reflects vitamin D status. Their joint association with paediatric ADHD remains incompletely characterised.

**Methods:**

In this retrospective case-control study, 78 treatment-naïve children with ADHD and 40 non-ADHD outpatient comparison children selected using frequency matching by age and sex were included. The most recent laboratory results available in the outpatient record were extracted. Given that some 25(OH)D values were derived from reports obtained outside our hospital, all available 25(OH)D records were manually re-checked against source documents and standardised to ng/mL prior to analysis. Group comparisons, whole-sample Spearman correlation analyses, logistic regression models including an interaction term between standardised SII and standardised 25(OH)D and exploratory receiver operating characteristic (ROC) analyses were performed. Sensitivity analysis and Firth penalised logistic regression were additionally conducted.

**Results:**

Compared with the comparison group, children with ADHD had significantly higher SII, C-reactive protein (CRP), neutrophil-to-lymphocyte ratio (NLR) and platelet-to-lymphocyte ratio (PLR) levels, together with lower serum 25(OH)D. The ADHD group was more likely to fall into lower clinically defined 25(OH)D categories, whereas the comparison group was more frequently in the ≥30 ng/mL category; this pattern remained directionally consistent after stratification by season of blood draw. In the whole study sample, SII was positively correlated with attention, impulsivity and oppositional scores, whereas serum 25(OH)D was inversely correlated with these symptom dimensions; SII was also inversely correlated with 25(OH)D. In the parsimonious model, the interaction term between standardised SII and standardised 25(OH)D was statistically significant (odds ratio (OR) = 3.06, 95% confidence interval (CI) 1.59–6.80, *p* = 0.002), and it remained significant in the broader sensitivity model and Firth penalised regression. In exploratory within-sample analysis, ROC curves yielded area under curves (AUCs) of 0.808 for SII, 0.781 for serum 25(OH)D and 0.836 for the parsimonious multivariable model.

**Conclusions:**

Paediatric ADHD was associated with high inflammatory burden and low serum 25(OH)D status in this retrospective outpatient sample. The statistically significant interaction between SII and 25(OH)D suggests a stable pattern of effect modification on the logistic model scale. These findings support a potentially relevant immuno-nutritional pattern in ADHD, but prospective validation is needed before causal interpretation or clinical implementation can be considered.

## Introduction

Attention-deficit/hyperactivity disorder (ADHD) is a prevalent neurodevelopmental condition originating in childhood. ADHD is characterised by persistent patterns of inattention, hyperactivity and impulsivity that exceed developmental norms. Recent evidence indicates that the pooled global prevalence of ADHD in children and adolescents is approximately 7.6%, while a recent national survey in China reported a prevalence of 6.4% among school-attending children aged 6–16 years [1, 2]. ADHD often persists into adulthood when left unaddressed; this persistence precipitates academic failure, occupational instability and severe social dysfunction, which impose a substantial socioeconomic and psychological burden [3, 4]. While progress has been made in understanding genetic susceptibility, neurotransmitter (dopamine and norepinephrine) dysregulation and delayed development of specific brain regions, the high clinical heterogeneity of ADHD suggests that critical pathological links remain unelucidated [5].

The “neuroinflammation hypothesis” has recently gained traction in explaining the pathophysiology of neurodevelopmental disorders. Peripheral inflammatory markers, such as the neutrophil-to-lymphocyte ratio (NLR) and platelet-to-lymphocyte ratio (PLR), have been investigated as potential biomarkers in several psychiatric conditions, including ADHD [6]. The systemic immune-inflammation index (SII) is a composite marker integrating neutrophil, platelet and lymphocyte counts and may better reflect systemic inflammatory burden than a single ratio [7]. Emerging evidence suggests that low-grade chronic peripheral inflammation may impair blood–brain barrier integrity, activate microglia and disrupt neural networks related to executive function [8]. However, the distribution of SII in paediatric ADHD, particularly in relation to symptom dimensions and subtype patterns, remains insufficiently characterised.

In parallel, vitamin D has attracted interest because of its neurodevelopmental and immunomodulatory relevance. The vitamin D receptor is expressed in several brain regions implicated in ADHD, including the prefrontal cortex, striatum and substantia nigra [9]. Beyond regulating neurotrophic factor synthesis, vitamin D may also contribute to neuroimmune homeostasis by modulating pro-inflammatory pathways [10, 11]. Low serum 25-hydroxyvitamin D (25(OH)D) levels have been reported in some ADHD populations [12, 13], but whether reduced 25(OH)D is an independent feature, a correlate of broader inflammatory burden or part of a more complex inflammation–nutrition pattern is still unclear [14, 15].

Using retrospective outpatient clinical data from a hospital in China, this study aimed to explore the relationship between inflammatory burden and vitamin D status in children with ADHD. By comparing the levels of SII and serum 25(OH)D between the ADHD and non-ADHD groups, we describe the clinical definition of 25(OH)D stratified distribution, explore its association with symptom indicators and evaluate the statistical interaction between SII and 25(OH)D in ADHD status. We hypothesised that ADHD is associated with high inflammatory load and low 25(OH)D status, and the two factors may not be independent.

## Materials and Methods

### Study Design and Ethics

This single-centre, retrospective case-control study was conducted in accordance with the Declaration of Helsinki and received approval from the Ethics Committee of Dongyang Maternal and Child Health Hospital (ethical approval number: 2026-LWS-04). Written informed consent was obtained from the parents of all participants. The clinical data used were de-identified and anonymised. These data were collected during routine care without any study intervention. All data handling complied with relevant ethical standards.

### Participants

We retrospectively screened consecutively available outpatient records from March 2024 to September 2025. The ADHD group comprised 78 treatment-naïve children aged 6–12 years who were newly diagnosed with ADHD during routine outpatient assessment. The comparison group comprised 40 outpatient children selected using frequency matching by age and sex from the same developmental-behavioural clinic who were evaluated during the same period for non-ADHD developmental, learning or minor behavioural concerns, as summarised in **Supplementary Table 1**. These children did not meet the Swanson, Nolan and Pelham Rating Scale-IV (SNAP-IV) screening threshold for ADHD and had no clinician-documented ADHD diagnosis after clinical evaluation. They were included as a clinical outpatient comparison group rather than as strictly healthy community comparison group. Laboratory testing was performed as part of routine outpatient evaluation according to clinical judgment (Fig. 1).

**Fig. 1.**
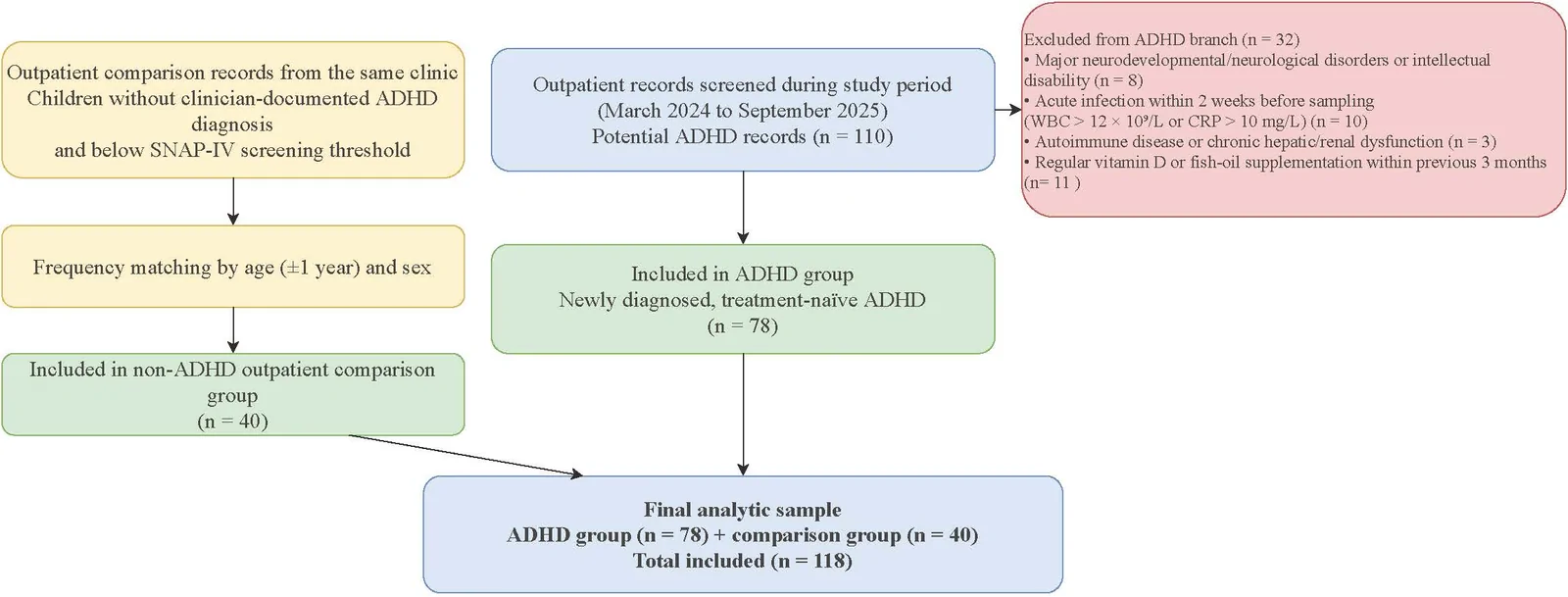
**Flow diagram of participant screening, exclusion, matching and final inclusion potential ADHD outpatient records were screened retrospectively**. Exclusions were applied sequentially according to predefined criteria. The comparison group consisted of non-ADHD outpatient children from the same clinic and period, who were selected using frequency matching by age (±1 year) and sex.

Inclusion Criteria: (1) Age 6–12 years. (2) ADHD diagnosis established according to diagnostic and statistical manual of mental disorders-5th ed (DSM-5) criteria during routine clinical assessment and reviewed by two senior developmental-behavioural paediatricians, with specified subtypes (predominantly inattentive presentation (ADHD-I), predominantly hyperactive/impulsive presentation (ADHD-HI), or combined presentation (ADHD-C)) [16]. (3) Treatment-naïve status at the time of initial diagnosis. (4) Availability of complete SNAP-IV data and relevant laboratory results in the outpatient record.

For research quantification, ADHD symptom dimensions were assessed using the SNAP-IV rating scale [17, 18]. Each SNAP-IV item was scored on a 4-point scale from 0 to 3, corresponding to “not at all”, “just a little”, “quite a bit”, and “very much”. Consistent with previous SNAP-IV screening applications, item scores of 2 or 3 were operationally treated as symptom-level endorsement for symptom-count purposes [18]. A symptom-count threshold of ≥6 endorsed symptoms within either the inattention or hyperactivity–impulsivity domain was used to correspond to the DSM-5 symptom domains [16].

Exclusion Criteria: (1) Comorbid neurodevelopmental (e.g. autism spectrum disorder), major neurological disorders or intellectual disability. (2) Acute infection within 2 weeks prior to sampling (WBC > 12 × 10^9^/L or C-reactive protein (CRP) > 10 mg/L) [19]. (3) Autoimmune diseases or chronic hepatic/renal dysfunction. (4) Regular vitamin D or fish-oil supplementation within the preceding 3 months.

Children in the comparison group also had no chart-documented history of major neurodevelopmental disorders, major neurological disease or severe chronic systemic illness at the time of selection. Given that this research was a retrospective study based on consecutively available eligible outpatient records, no formal a priori sample size calculation was performed.

### Data Collection and Variable Definitions

Demographic and clinical variables included age, sex, height, weight, body mass index (BMI), season of blood draw, parental education level, residence type, oppositional defiant disorder (ODD) comorbidity, emotional issues record and allergic disease history.

ODD comorbidity was defined according to the corresponding clinician-documented diagnosis in the medical record at the time of assessment. Record of emotional issues was defined as chart-documented emotional concerns noted during routine outpatient evaluation, which was based on the medical record of the treating clinician rather than a separate standardised psychiatric diagnostic interview. Allergic disease history was defined as parent-reported or chart-documented history of allergic conditions recorded in the medical file.

Behavioural symptoms were assessed using the parent-rated Chinese version of the SNAP-IV. The Chinese SNAP-IV Parent Form has been validated in school-aged Chinese populations and has shown satisfactory internal consistency, with Cronbach’s alpha coefficients of approximately 0.88–0.90 across symptom dimensions, as well as acceptable psychometric performance [20, 21]. Internal consistency was not re-estimated in the present sample because this study was a retrospective analysis based on archived clinical records. The scale includes the inattention (9 items), hyperactivity/impulsivity (9 items) and oppositional defiance (8 items) dimensions. Items were scored 0–3, with dimension scores representing the mean of corresponding items.

### Laboratory Measurements and Biomarker Handling

Laboratory variables included neutrophil count, lymphocyte count, platelet count, CRP, SII, NLR, PLR and serum 25(OH)D. CRP was recorded in mg/L. SII was calculated as (platelet count × neutrophil count)/lymphocyte count [22]. NLR was calculated as neutrophil count/lymphocyte count, and PLR as platelet count/lymphocyte count.

For this retrospective outpatient study, the most recent laboratory results available in the outpatient record at the time of clinical assessment were extracted. These values were not necessarily generated within our hospital; for some children, laboratory results were based on reports obtained from outside institutions and manually entered into the outpatient medical record. Considering that serum 25(OH)D data could originate from reports obtained at different institutions, all available 25(OH)D values were manually re-checked against the source records and standardised to ng/mL before analysis. Accordingly, 37 of 118 participants (31.4%) had at least one relevant laboratory result derived from outside institutions. For clinical interpretation, serum 25(OH)D was analysed as a continuous variable and as a categorical variable using the following strata: <20, 20–29.9 and ≥30 ng/mL.

### Statistical Analysis

All statistical analyses were performed using R version 4.3.0 (R Foundation for Statistical Computing, Vienna, Austria). Continuous variables were expressed as median (interquartile range), and categorical variables as number (percentage). Between-group comparisons were performed using the Wilcoxon rank-sum test, Pearson’s chi-square test or Fisher’s exact test, as appropriate. Multi-group comparisons within ADHD subtypes were performed using one-way analysis of variance (ANOVA), the Kruskal–Wallis test, or Fisher’s exact test, as appropriate.

In addition to continuous analyses, the categorical distribution of clinically defined 25(OH)D strata was compared between groups and further examined within each season of blood draw. CRP was also categorised descriptively (<5 and 5–10 mg/L) to facilitate interpretation of its distribution within the non-acute inflammatory range.

Spearman correlation analyses were performed across the whole study sample. Given that these analyses were based on the combined ADHD-comparison sample, they were intended to describe cross-sample gradients rather than within-ADHD severity associations. SII and serum 25(OH)D were treated as the primary biomarkers of interest. Analyses involving NLR, PLR, CRP distribution, subtype comparisons and ROC curves were considered exploratory and were interpreted accordingly.

To evaluate the joint association of inflammatory burden and vitamin D status with ADHD case status, multivariable logistic regression models including an interaction term between standardised SII and standardised 25(OH)D were fitted. SII and 25(OH)D were transformed to z-scores before model entry. A parsimonious primary model adjusted for age, sex, BMI and season of blood draw. A broader sensitivity model further adjusted for parental education level, residence type and CRP. Firth penalised logistic regression was performed as a robustness analysis. To evaluate the potential influence of mixed-source laboratory data, we additionally performed a sensitivity analysis restricted to this internal-only subset.

Predicted probabilities of ADHD were generated from the parsimonious model across clinically defined 25(OH)D strata and SII percentiles (25th, 50th and 75th). ROC analysis was used only as an exploratory assessment of within-sample discrimination rather than clinical diagnostic performance.

Model diagnostics included variance inflation factors (VIFs), the Hosmer–Lemeshow goodness-of-fit test and influence diagnostics. Missingness was minimal in the key analytic variables; therefore, complete-case analysis was used. Given that subtype analyses were secondary and exploratory, an approximate post-hoc power analysis based on the observed effect size for the SII subtype comparison was additionally performed. A two-sided *p* < 0.05 was considered statistically significant.

## Results

### Baseline Characteristics

The study included 118 subjects (ADHD: 78; comparison group: 40). Frequency matching resulted in comparable demographic profiles (Table 1). No significant differences were observed in age (*p* = 0.359), sex (*p* = 0.702), BMI (*p* = 0.845), sampling season, parental education or residence type (all *p* > 0.05). As shown in **Supplementary Table 1**, most comparison children (87.5%) were evaluated for attention-related or learning/developmental concerns that did not meet ADHD diagnostic criteria, whereas only a small proportion (12.5%) presented with minor non-specific complaints.

**Table 1.** **Comparison of demographic characteristics between the ADHD and Comparison groups**.

Variables		Total (n = 118)	ADHD (n = 78)	Comparison (n = 40)	Statistic	*p*
Age (years), M (Q_1_, Q_3_)		8.90 (7.42, 10.50)	8.64 (7.22, 10.47)	9.18 (7.78, 10.45)	Z = -0.92	0.359
Height (cm), M (Q_1_, Q_3_)		135.36 (124.55, 147.09)	134.90 (122.69, 147.02)	136.10 (125.13, 147.40)	Z = -0.61	0.541
Weight (kg), M (Q_1_, Q_3_)		33.70 (27.41, 40.71)	34.38 (27.42, 40.71)	32.13 (27.45, 40.64)	Z = -0.21	0.836
BMI (kg/m^2^), M (Q_1_, Q_3_)		19.27 (14.35, 22.61)	19.33 (14.41, 22.41)	19.18 (13.61, 22.74)	Z = -0.20	0.845
Sex, n (%)					χ^2^ = 0.15	0.702
	Female	56 (47.46)	38 (48.72)	18 (45.00)		
	Male	62 (52.54)	40 (51.28)	22 (55.00)		
Season of blood draw, n (%)					χ^2^ = 0.99	0.803
	Autumn	32 (27.12)	22 (28.21)	10 (25.00)		
	Spring	22 (18.64)	13 (16.67)	9 (22.50)		
	Summer	34 (28.81)	24 (30.77)	10 (25.00)		
	Winter	30 (25.42)	19 (24.36)	11 (27.50)		
Parental education level, n (%)					χ^2^ = 5.00	0.172
	Bachelor degree	60 (50.85)	41 (52.56)	19 (47.50)		
	High school	32 (27.12)	24 (30.77)	8 (20.00)		
	Junior high school or below	16 (13.56)	9 (11.54)	7 (17.50)		
	Master’s degree or above	10 (8.47)	4 (5.13)	6 (15.00)		
Residence type, n (%)					χ^2^ = 1.67	0.434
	Rural	27 (22.88)	18 (23.08)	9 (22.50)		
	Suburban	22 (18.64)	17 (21.79)	5 (12.50)		
	Urban	69 (58.47)	43 (55.13)	26 (65.00)		

Notes: Data are presented as median (Q_1_, Q_3_) or n (%). *p* values were calculated using the Mann–Whitney U test for continuous variables and the chi-square test for categorical variables, as appropriate. BMI, body mass index; M, median; Q_1_, first quartile; Q_3_, third quartile; Z, Mann–Whitney U test; χ^2^, chi-square test; ADHD, attention-deficit/hyperactivity disorder.

As shown in Table 2, children with ADHD exhibited significantly higher inflammatory markers, including neutrophil counts, platelet counts, CRP, SII, NLR and PLR (all *p* < 0.05). Notably, SII levels were markedly elevated in the ADHD group compared with those in comparison group (698.58 (478.56, 925.51) vs. 424.98 (270.04, 550.02), *p* < 0.001), as were NLR and PLR. Serum 25(OH)D was significantly lower in ADHD cases (28.96 (27.92, 29.71) vs. 30.11 (29.67, 40.99), *p* < 0.001). CRP was also higher in the ADHD group (8.88 (4.89, 9.37) vs. 5.12 (3.93, 6.99), *p* < 0.001), although all included participants remained below the predefined exclusion threshold of 10 mg/L. ADHD was also associated with higher rates of ODD comorbidity, emotional issues and allergic history (*p* < 0.001).

**Table 2.** **Comparisons of inflammatory markers, CRP distribution, 25(OH)D status and clinical scores between the ADHD and comparison group**.

Variables		Total (n = 118)	ADHD (n = 78)	Comparison (n = 40)	Statistic	*p*
Attention score, M (Q_1_, Q_3_)		1.11 (0.59, 2.11)	1.89 (1.11, 2.33)	0.56 (0.33, 0.70)	Z = -7.79	<0.001
Impulsivity score, M (Q_1_, Q_3_)		0.83 (0.56, 2.41)	2.22 (0.78, 2.56)	0.56 (0.44, 0.67)	Z = -6.71	<0.001
Oppositional score, M (Q_1_, Q_3_)		1.13 (0.63, 1.75)	1.50 (1.13, 2.00)	0.50 (0.38, 0.72)	Z = -8.29	<0.001
Neutrophil (10^9^/L), M (Q_1_, Q_3_)		5.37 (3.94, 6.62)	5.66 (4.48, 6.77)	4.30 (3.26, 6.18)	Z = -2.14	0.032
Lymphocyte (10^9^/L), M (Q_1_, Q_3_)		2.46 (1.77, 3.26)	2.20 (1.59, 2.95)	2.66 (1.96, 3.65)	Z = -2.81	0.005
Platelet (10^9^/L), M (Q_1_, Q_3_)		283.02 (225.76, 327.17)	293.71 (249.76, 338.55)	233.82 (206.03, 285.03)	Z = -3.31	<0.001
CRP (mg/L), M (Q_1_, Q_3_)		6.94 (4.22, 9.37)	8.88 (4.89, 9.37)	5.12 (3.93, 6.99)	Z = -3.75	<0.001
CRP levels					-	0.039
	<5 mg/L, n (%)	40(33.90)	21 (26.92)	19 (47.50)		
	5–10 mg/L, n (%)	78(66.10)	57 (73.08)	21 (52.50)		
SII (10^9^/L), M (Q_1_, Q_3_)		568.79 (440.24, 817.49)	698.58 (478.56, 925.51)	424.98 (270.04, 550.02)	Z = -5.47	<0.001
NLR, M (Q_1_, Q_3_)		2.13 (1.60, 2.96)	2.43 (1.76, 3.44)	1.84 (1.24, 2.16)	Z = -3.87	<0.001
PLR, M (Q_1_, Q_3_)		112.34 (87.03, 161.75)	136.27 (91.80, 177.12)	89.98 (63.97, 114.50)	Z = -4.10	<0.001
25(OH)D (ng/mL), M (Q_1_, Q_3_)		29.45 (28.01, 30.20)	28.96 (27.92, 29.71)	30.11 (29.67, 40.99)	Z = -4.99	<0.001
25(OH)D status, n (%)					χ^2^ = 35.48	<0.001
	<20	14 (11.86)	11 (14.10)	3 (7.50)		
	20–29.9	64 (54.24)	55 (70.51)	9 (22.50)		
	≥30	40 (33.90)	12 (15.38)	28 (70.00)		
ODD comorbidity, n (%)					χ^2^ = 16.18	<0.001
	No	64 (54.24)	32 (41.03)	32 (80.00)		
	Yes	54 (45.76)	46 (58.97)	8 (20.00)		
Emotional issues record, n (%)					χ^2^ = 22.11	<0.001
	No	68 (57.63)	33 (42.31)	35 (87.50)		
	Yes	50 (42.37)	45 (57.69)	5 (12.50)		
Allergic disease history, n (%)					χ^2^ = 18.31	<0.001
	No	59 (50.00)	28 (35.90)	31 (77.50)		
	Yes	59 (50.00)	50 (64.10)	9 (22.50)		

Notes: Data are presented as median (Q_1_, Q_3_) or n (%). Neutrophil, lymphocyte and platelet counts are expressed as 10^9^/L; CRP is expressed as mg/L; 25(OH)D is expressed as ng/mL. SII was calculated as platelet count × neutrophil count/lymphocyte count. CRP, C-reactive protein; SII, systemic immune-inflammation index; NLR, neutrophil-to-lymphocyte ratio; PLR, platelet-to-lymphocyte ratio; 25(OH)D, 25-hydroxyvitamin D; ODD, oppositional defiant disorder; M, median; Q_1_, first quartile; Q_3_, third quartile; ADHD, attention-deficit/hyperactivity disorder; Z, Mann–Whitney U test; χ^2^, chi-square test; -, Fisher’s exact test.

Categorical analysis of serum 25(OH)D showed a marked between-group difference. In the ADHD group, 11/78, 55/78 and 12/78 children were classified as <20, 20–29.9 and ≥30 ng/mL, respectively, compared with 3/40, 9/40 and 28/40 in the control group (*p* < 0.001; Table 2). Thus, children with ADHD were more likely to fall into lower 25(OH)D categories, whereas comparison group were more frequently in the ≥30 ng/mL category. When stratified by season of blood draw, this pattern remained directionally consistent across spring, summer, autumn and winter (all Fisher’s exact *p* < 0.05; **Supplementary Table 2**). Therefore, the observed between-group difference was not solely attributable to seasonal imbalance.

In the whole study sample, Spearman correlation analysis showed that SII was positively correlated with attention score (rho = 0.356), impulsivity score (rho = 0.450) and oppositional score (rho = 0.453). By contrast, serum 25(OH)D was inversely correlated with attention score (rho = -0.312), impulsivity score (rho = -0.310) and oppositional score (rho = -0.290). SII was also inversely correlated with serum 25(OH)D (rho = -0.457) (Fig. 2). Considering that these analyses were performed in the combined ADHD-comparison sample, the observed correlations should be interpreted primarily as cross-sample gradients rather than within-ADHD severity associations.

**Fig. 2.**
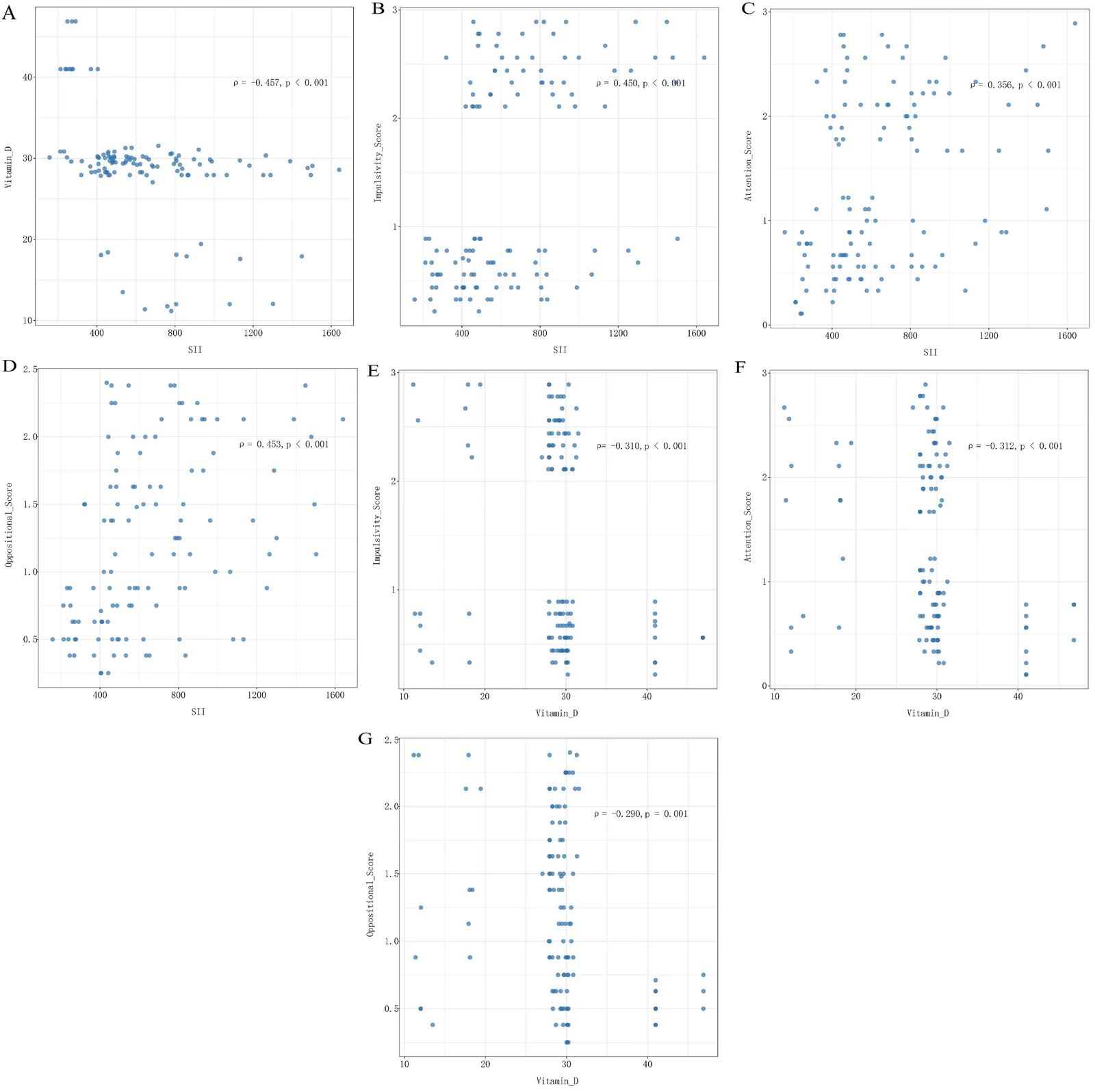
**Correlations among SII, 25(OH)D and SNAP-IV symptom scores**. SNAP-IV, Swanson, Nolan and Pelham Rating Scale-IV; SII, systemic immune-inflammation index; 25(OH)D, 25-hydroxyvitamin D. (A) Scatter plot showing the correlation between SII and 25(OH)D levels (Spearman’s r = -0.457). (B) Correlation between SII and Impulsivity_Score (r = 0.450). (C) Correlation between SII and Attention_Score (r = 0.356). (D) Correlation between SII and Oppositional_Score (r = 0.453). (E) Correlation between 25(OH)D and Impulsivity_Score (r = -0.310). (F) Correlation between 25(OH)D and Attention_Score (r = -0.312). (G) Correlation between 25(OH)D and Oppositional_Score (r = -0.290).

Among the 78 children with ADHD, the subtype distribution was 28 for ADHD-C, 23 for ADHD-HI and 27 for ADHD-I. Significant subtype differences were observed in platelet count (*p* = 0.037), as well as in attention, hyperactivity/impulsivity and oppositional symptom scores and counts (all *p* < 0.001). This finding is consistent with expected subtype-specific symptom profiles. However, no significant subtype differences were found in season of blood draw (*p* = 0.381), serum 25(OH)D (*p* = 0.992), CRP (*p* = 0.611), SII (*p* = 0.164), NLR (*p* = 0.113) or PLR (*p* = 0.393) (Table 3). Although no significant subtype difference in SII was observed (*p* = 0.164), the approximate post-hoc power based on the observed effect size was only 0.280, which indicates limited capability to detect modest between-subtype differences.

**Table 3.** **Differences in clinical and laboratory variables among ADHD subtypes**.

Variables		Total (n = 78)	ADHD-C (n = 28)	ADHD-HI (n = 23)	ADHD-I (n = 27)	Statistic	*p*
Platelet (10^9^/L), Mean ± SD		294.08 ± 62.51	311.42 ± 66.72	267.52 ± 59.23	298.74 ± 54.88	F = 3.43	0.037
Attention score, M (Q_1_, Q_3_)		1.89 (1.11, 2.33)	2.33 (2.19, 2.67)	0.89 (0.67, 1.11)	1.89 (1.75, 2.05)	H = 50.05	<0.001
Attention count, M (Q_1_, Q_3_)		6.00 (3.00, 8.00)	8.00 (7.00, 9.00)	2.00 (1.00, 3.00)	6.00 (6.00, 7.00)	H = 48.87	<0.001
Impulsivity score, M (Q_1_, Q_3_)		2.22 (0.78, 2.56)	2.44 (2.22, 2.56)	2.44 (2.28, 2.72)	0.67 (0.50, 0.78)	H = 47.75	<0.001
Impulsivity count, M (Q_1_, Q_3_)		7.00 (2.00, 8.00)	8.00 (7.00, 8.00)	8.00 (7.50, 9.00)	1.00 (0.50, 2.00)	H = 50.92	<0.001
Oppositional score, M (Q_1_, Q_3_)		1.50 (1.13, 2.00)	2.13 (2.00, 2.25)	1.50 (1.38, 1.63)	1.00 (0.88, 1.25)	H = 45.06	<0.001
Oppositional count, M (Q_1_, Q_3_)		3.00 (2.00, 6.00)	6.00 (6.00, 7.00)	3.00 (3.00, 4.00)	2.00 (1.00, 3.00)	H = 45.72	<0.001
Neutrophil (10^9^/L), M (Q_1_, Q_3_)		5.66 (4.48, 6.77)	5.94 (4.36, 6.83)	5.74 (4.71, 7.09)	5.39 (4.58, 5.93)	H = 1.28	0.528
Lymphocyte (10^9^/L), M (Q_1_, Q_3_)		2.20 (1.59, 2.95)	2.27 (1.51, 2.78)	2.03 (1.34, 2.55)	2.62 (1.81, 3.22)	H = 3.82	0.148
CRP (mg/L), M (Q_1_, Q_3_)		8.88 (4.89, 9.37)	8.92 (5.46, 9.37)	9.37 (4.77, 9.37)	7.20 (5.21, 9.37)	H = 0.98	0.611
SII (10^9^/L), M (Q_1_, Q_3_)		698.58 (478.56, 925.51)	770.41 (563.11, 944.31)	709.54 (489.88, 944.82)	645.76 (441.60, 815.85)	H = 3.62	0.164
NLR, M (Q_1_, Q_3_)		2.43 (1.76, 3.44)	2.47 (2.12, 3.16)	2.57 (1.80, 3.93)	1.93 (1.65, 2.92)	H = 4.36	0.113
PLR, M (Q_1_, Q_3_)		136.27 (91.80, 177.12)	135.12 (104.48, 191.78)	137.01 (91.47, 179.02)	112.31 (89.71, 158.92)	H = 1.87	0.393
Season of blood draw, n (%)						-	0.381
	Autumn	22 (28.21)	4 (14.29)	9 (39.13)	9 (33.33)		
	Spring	13 (16.67)	7 (25.00)	3 (13.04)	3 (11.11)		
	Summer	24 (30.77)	11 (39.29)	5 (21.74)	8 (29.63)		
	Winter	19 (24.36)	6 (21.43)	6 (26.09)	7 (25.93)		
25(OH)D (ng/mL), M (Q_1_, Q_3_)		28.96 (27.92, 29.71)	28.69 (27.89, 29.89)	28.43 (27.92, 29.59)	29.06 (27.92, 29.62)	H = 0.02	0.992
25(OH)D status, n (%)						-	0.881
	<20	11 (14.10)	5 (17.86)	2 (8.70)	4 (14.81)		
	20–29.9	55 (70.51)	18 (64.29)	18 (78.26)	19 (70.37)		
	≥30	12 (15.38)	5 (17.86)	3 (13.04)	4 (14.81)		

Notes: Data are presented as mean ± SD, median (Q_1_, Q_3_) or n (%). Neutrophil, lymphocyte and platelet counts are expressed as 10^9^/L; CRP is expressed as mg/L; 25(OH)D is expressed as ng/mL. ADHD, attention-deficit/hyperactivity disorder; ADHD-C, combined presentation; ADHD-HI, predominantly hyperactive/impulsive presentation; ADHD-I, predominantly inattentive presentation; CRP, C-reactive protein; SII, systemic immune-inflammation index; NLR, neutrophil-to-lymphocyte ratio; PLR, platelet-to-lymphocyte ratio; 25(OH)D, 25-hydroxyvitamin D; M, median; Q_1_, first quartile; Q_3_, third quartile; SD, standard deviation; F, one-way analysis of variance; H, Kruskal-Wallis test; -, Fisher’s exact test.

Multivariable logistic regression models including an interaction term between standardised SII and standardised 25(OH)D are shown in Table 4. In the parsimonious model, standardised SII was significantly associated with ADHD status (OR = 6.94, 95% CI 2.77–22.64, *p* < 0.001), whereas the main effect of standardised 25(OH)D was not statistically significant (OR = 0.82, 95% CI 0.33–1.69, *p* = 0.617). Importantly, the interaction term between standardised SII and standardised 25(OH)D was statistically significant (OR = 3.06, 95% CI 1.59–6.80, *p* = 0.002). This interaction remained significant in the broader sensitivity model (OR = 4.52, 95% CI 2.01–12.91, *p* = 0.001) and in Firth penalised logistic regression (OR = 3.18, 95% CI 1.64–7.72, *p* < 0.001). Multicollinearity was low (all ${\mathrm{GVIF}}^{\left(1/2\,D\,f\right)}$ < 1.6, where GVIF is generalized variance inflation factor, Df is degrees of freedom), calibration was acceptable (Hosmer–Lemeshow *p* > 0.05) and influence diagnostics did not materially alter the main conclusions. These findings support a stable pattern of effect modification on the logistic model scale.

**Table 4.** **Logistic regression models including the interaction term between standardised SII and standardised 25(OH)D**.

Variable	Parsimonious model OR (95% CI)	*p*	Sensitivity model OR (95% CI)	*p*	Firth model OR (95% CI)	*p*
Standardised SII	6.94 (2.77–22.64)	<0.001	7.05 (2.39–28.43)	0.002	4.45 (1.78–14.85)	<0.001
Standardised 25(OH)D	0.82 (0.33–1.69)	0.617	0.63 (0.23–1.43)	0.306	0.72 (0.31–1.46)	0.373
SII × 25(OH)D	3.06 (1.59–6.80)	0.002	4.52 (2.01–12.91)	0.001	3.18 (1.64–7.72)	<0.001

Notes: The parsimonious model adjusted for age, sex, BMI and season of blood draw. The sensitivity model additionally adjusted for parental education, residence type and CRP. SII and 25(OH)D were entered as standardised z-scores. SII, systemic immune-inflammation index; CRP, C-reactive protein; BMI, body mass index; OR, odds ratio; CI, confidence interval; 25(OH)D, 25-hydroxyvitamin D.

In an additional sensitivity analysis restricted to participants whose serum 25(OH)D and complete blood count (CBC)-derived indices were both generated within our hospital (n = 81), the main findings remained broadly consistent. In the internal-only parsimonious model, standardised SII remained significantly associated with ADHD status (OR = 8.14, 95% CI 2.78–34.97, *p* = 0.001), whereas the main effect of standardised 25(OH)D remained non-significant (OR = 0.80, 95% CI 0.32–1.72, *p* = 0.585). Importantly, the interaction between standardised SII and standardised 25(OH)D remained statistically significant in the internal-only parsimonious model (OR = 3.03, 95% CI 1.46–7.53, *p* = 0.007), the broader internal-only sensitivity model (OR = 3.80, 95% CI 1.59–12.67, *p* = 0.009) and Firth penalised regression (OR = 2.49, 95% CI 1.26–6.48, *p* < 0.001) (**Supplementary Table 3**). These findings support that the main interaction pattern was not solely driven by mixed-source laboratory data.

Predicted-probability analysis based on clinically defined 25(OH)D strata and SII percentiles further illustrated the interaction pattern (Fig. 3). In the 20–29.9 ng/mL and ≥30 ng/mL strata, higher SII percentiles were associated with higher predicted probabilities of ADHD. By contrast, in the <20 ng/mL stratum, predicted probability remained uniformly high and did not show a further increase across SII percentiles. This pattern indicates that the association between inflammatory burden and ADHD case status varies according to 25(OH)D status, rather than reflecting a simple uniform synergistic increase. Given that the <20 ng/mL subgroup was relatively small, the predicted pattern in this stratum should be interpreted cautiously.

**Fig. 3.**
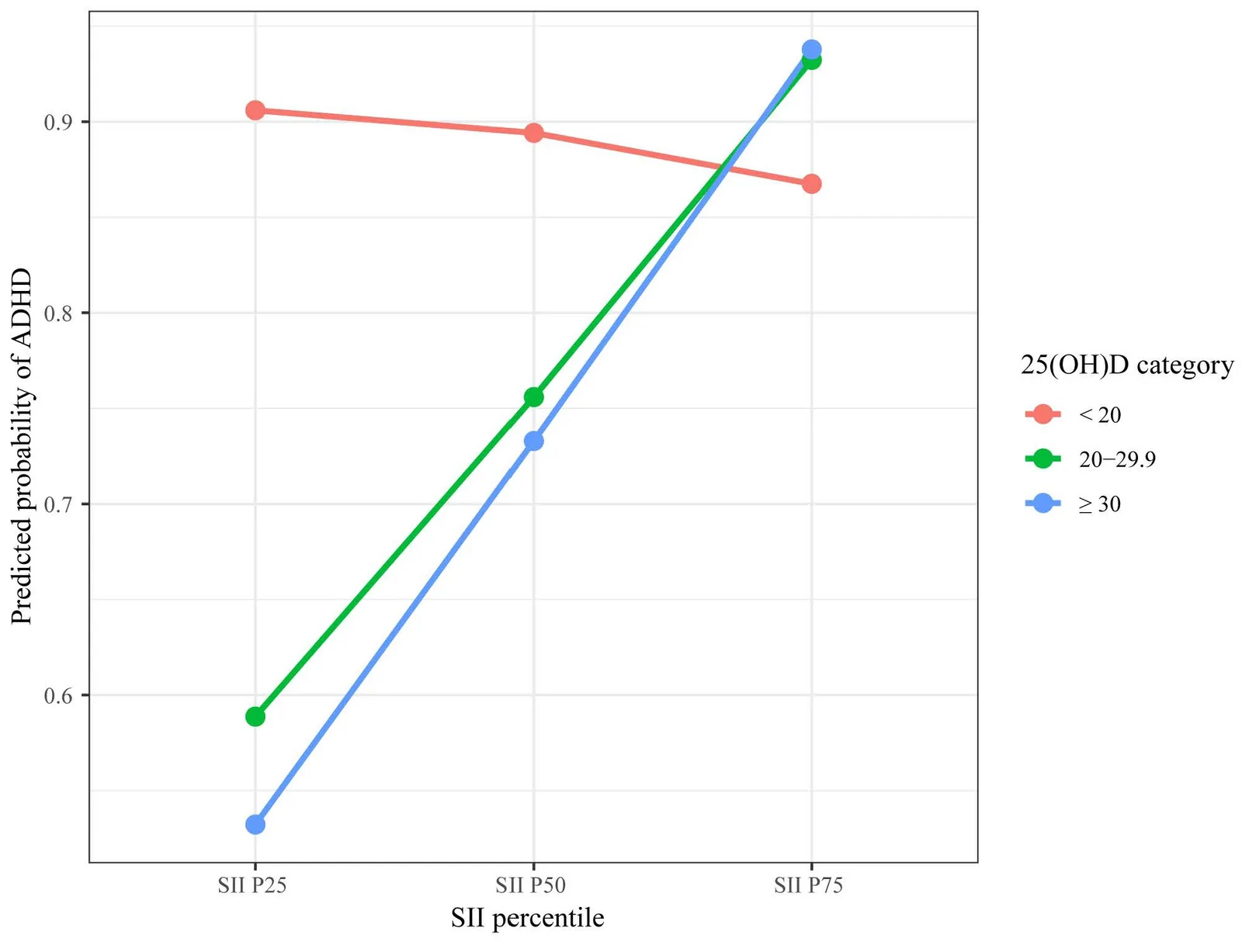
**Predicted probability of ADHD across clinically defined 25(OH)D strata and SII percentiles**. Predicted probabilities were generated from the parsimonious logistic regression model across clinically defined 25(OH)D strata (<20, 20–29.9 and ≥30 ng/mL) and SII percentiles (P25, P50 and P75). Estimates in the <20 ng/mL stratum should be interpreted with caution because of the small subgroup size. ADHD, attention-deficit/hyperactivity disorder; SII, systemic immune-inflammation index; 25(OH)D, 25-hydroxyvitamin D.

ROC analysis was performed as an exploratory assessment of within-sample discrimination rather than clinical diagnostic performance. SII yielded an area under curve (AUC) of 0.81, serum 25(OH)D yielded an AUC of 0.78 and the parsimonious multivariable model yielded an AUC of 0.84 (Fig. 4). These findings suggest moderate discrimination within the present dataset, with slightly improved performance for the combined model.

**Fig. 4.**
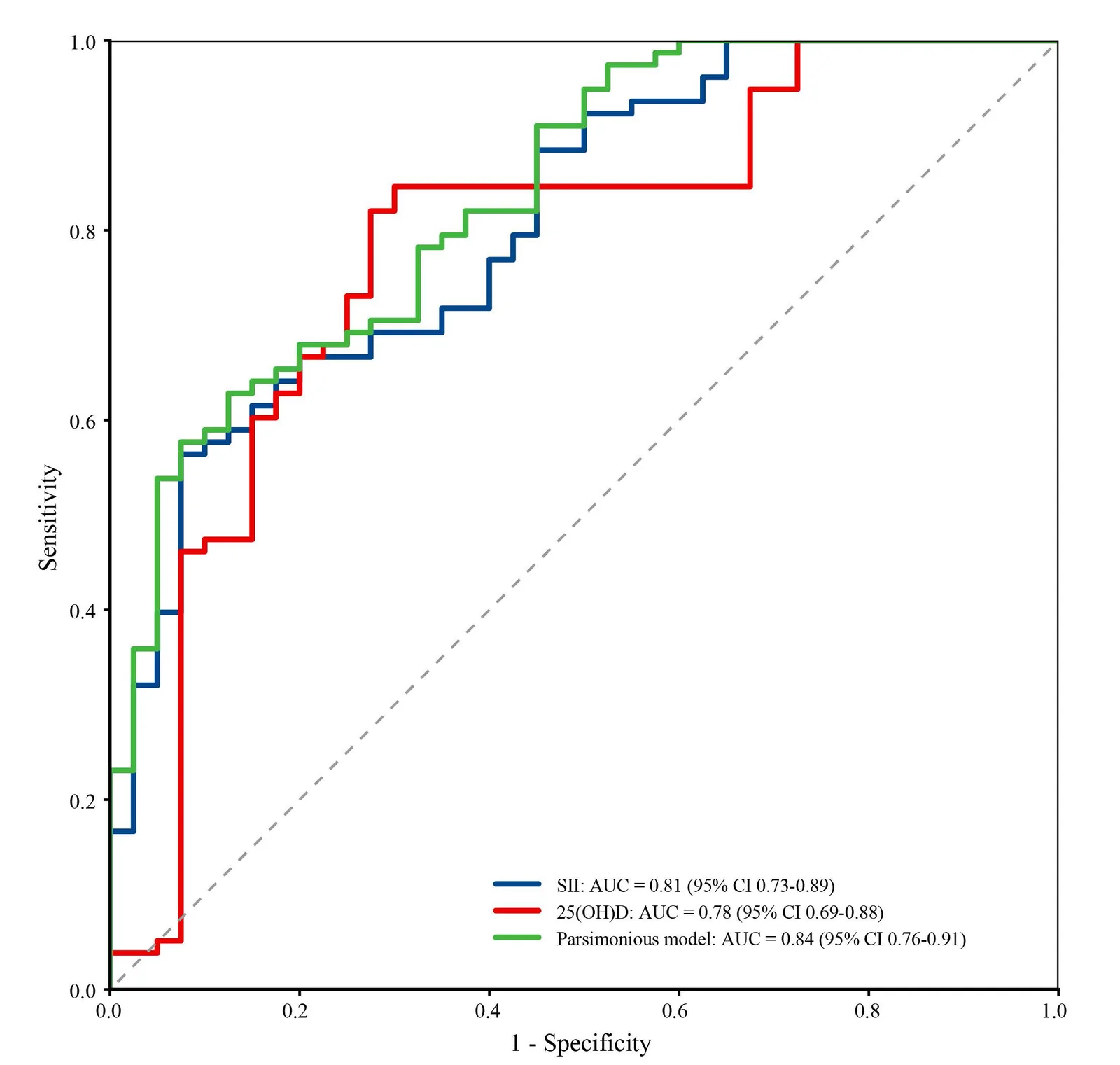
**ROC curves for SII, serum 25(OH)D and the parsimonious multivariable model in relation to ADHD case status**. ROC analysis is presented as an exploratory assessment of within-sample discrimination rather than clinical diagnostic performance. SII and serum 25(OH)D are shown as single markers, and the parsimonious model represents their combined discrimination after incorporation of the interaction term and key covariates. ADHD, attention-deficit/hyperactivity disorder; SII, systemic immune-inflammation index; ROC, receiver operating characteristic; 25(OH)D, 25-hydroxyvitamin D; AUC, area under curve; CI, confidence interval.

## Discussion

This retrospective outpatient study investigated inflammatory burden and serum 25(OH)D status in children with ADHD. The results showed that ADHD children had high SII, CRP, NLR and PLR, and low serum 25(OH)D levels. In addition to the inter-group differences in continuous variables, the ADHD group was more likely to be distributed in the lower 25(OH)D stratification, and it remained consistent after stratification by blood collection season. In the whole sample, higher SII and lower 25(OH)D were associated with higher symptom scores, and the interaction between SII and 25(OH)D remained statistically significant in multiple regression models.

### SII and Peripheral Inflammatory Burden

As a composite marker of immune activation, SII showed a correlation with ADHD, which aligns with findings by Schumacher *et al*. [23] in adolescent depression. Given that SII integrates neutrophils, platelets and lymphocytes, it may reflect a broader pattern of peripheral inflammatory burden than isolated single-cell indices. In neuroinflammatory settings, neutrophils can release cytokines, reactive oxygen species and matrix metalloproteinases such as matrix metalloproteinase-9 (MMP-9), which may contribute to blood–brain barrier dysfunction [24, 25]. In addition, platelets are closely linked to serotonin biology because they take up serotonin from plasma via the serotonin transporter, store it in dense granules and release it upon activation. Therefore, platelet-related indices may partly reflect broader neuroimmune and serotonergic processes, although their direct relationship with specific ADHD symptom dimensions remains to be clarified [26, 27]. Notably, the present findings should be interpreted as supportive of an inflammatory association rather than as proof of a specific mechanistic pathway. In addition, although some subtype differences were observed for platelet counts, the subtype analyses were underpowered for several biomarker comparisons and should therefore be interpreted cautiously. CRP was also high in the ADHD group, which supports the presence of a broader low-grade inflammatory background beyond CBC-derived indices. However, considering that all included participants had CRP values below 10 mg/L, this finding should be interpreted as a non-acute inflammatory shift rather than evidence of overt infection.

### 25(OH)D Status in ADHD

The inverse correlation between 25(OH)D and core symptom scores, particularly inattention, may be compatible with a role for 25(OH)D in dopaminergic regulation, although this interpretation remains indirect [28]. Low levels of 25(OH)D may also be associated with reduced neurodevelopment and weakened resistance to oxidative stress and neuroinflammation [29, 30, 31]. However, the results of this study on 25(OH)D should be interpreted with caution. After source-record verification and unit standardisation, serum 25(OH)D remained lower in the ADHD group than in the comparison group, but its main feature was not a clear absence in all cases, but that ADHD children as a whole were more likely to be distributed in the lower 25(OH)D stratification. In the whole sample, the negative correlation between 25(OH)D and symptom score was consistent with a broader “inflammation–nutrition” pattern, but it should not be interpreted as a linear association of symptom severity within the ADHD group.

### Interaction Between SII and 25(OH)D

A key finding of this study was the statistically significant interaction between SII and 25(OH)D in relation to ADHD case status. Importantly, this interaction remained significant in the parsimonious model, the broader sensitivity model and Firth penalised regression, which suggests a stable pattern of effect modification on the logistic model scale. Predicted-probability analysis further showed that the relationship between SII percentile and ADHD probability differed across clinically defined 25(OH)D strata. However, this pattern was not uniformly monotonic across all strata, particularly in the relatively small <20 ng/mL subgroup. Therefore, the present findings are better interpreted as indicating that the inflammatory association with ADHD varies according to 25(OH)D status, rather than as proof of a simple or fully established biological synergy. Previous studies have suggested that vitamin D may be relevant to ADHD through immunomodulatory, oxidative stress-related and neurotransmitter-related pathways [32, 33]. However, these mechanisms were not directly examined in the present study and are mentioned only as possible biological context.

### Clinical Implications and Future Directions

The ROC results in this study are only exploratory descriptions. Considering that the analysis is based on retrospective case-control data and lacks external validation, these results do not mean that they have clinical diagnostic performance and are insufficient to support the immediate formation of screening recommendations. From a clinical perspective, the results of this study suggest that ADHD may have an ‘inflammation–nutrition’-related model that deserves further attention. However, given that this research is a retrospective observational study, the current results should not be used directly to develop vitamin D supplementation programmes, anti-inflammatory intervention strategies or biomarker-based screening pathways. In the future, prospective, preferably multi-centre studies are still needed to determine whether these indicators can provide information with real application value in addition to routine clinical evaluation.

### Limitations

Several limitations should be acknowledged. First, this was a retrospective single-centre outpatient study, which limits causal inference and may introduce selection bias. In addition, region-specific environmental and clinical practice factors may limit the generalisability of the findings. Second, the comparison group consisted of non-ADHD outpatient comparison children rather than strictly healthy community comparison group. As summarised in **Supplementary Table 1**, most comparison children presented with developmental or learning-related concerns, whereas a smaller proportion had minor non-specific complaints. Therefore, their baseline inflammatory and nutritional profiles may not fully represent those of a truly healthy population. This clinical outpatient comparison structure may have introduced residual confounding, which should be regarded as an important methodological limitation when interpreting between-group biomarker differences. Accordingly, the between-group biomarker differences should be interpreted as comparisons against a clinical outpatient reference group rather than a strictly healthy population baseline. Third, some laboratory values, particularly serum 25(OH)D, were derived from reports obtained outside our hospital and manually entered into the outpatient record. Although source-record verification and unit standardisation were performed, inter-institutional heterogeneity in assay platform and reporting format cannot be fully excluded. Nevertheless, an additional internal-only sensitivity analysis showed that the main interaction pattern remained statistically consistent after restricting the analysis to within-hospital laboratory data. Fourth, considering that the study was conducted in an outpatient clinical setting, residual low-grade inflammatory conditions, including allergic inflammation or subclinical infection not captured by the exclusion criteria, cannot be completely excluded. Fifth, important determinants of 25(OH)D status, including sunlight exposure, outdoor activity and dietary intake, were not directly measured. In addition, allergic disease history and emotional issues record were based on chart documentation and may have been affected by recall or recording bias. Sixth, the correlation analyses were based on the combined ADHD-comparison sample and should therefore not be overinterpreted as within-ADHD severity relationships. Seventh, independently recorded pre-consensus diagnostic ratings were not systematically retained in this retrospective dataset, and formal inter-rater agreement statistics such as kappa could not be calculated. Lastly, the subtype analyses were limited by relatively small subgroup sample sizes and low approximate post-hoc power; therefore, null subtype findings should be interpreted cautiously.

## Conclusions

In this retrospective outpatient case-control study, paediatric ADHD was associated with high inflammatory burden and low serum 25(OH)D status. The interaction between SII and 25(OH)D remained statistically significant across multiple models, which supports a stable pattern of effect modification on the logistic model scale. These findings suggest a potentially relevant immuno-nutritional pattern in ADHD, but prospective validation is required before causal interpretation or clinical implementation can be considered.

## Availability of Data and Materials

The data that support the findings of this study are available from the corresponding author upon reasonable request.
