# Supplementary material for: Association Among Systemic Immune-Inflammation Index, Vitamin D Status and Clinical Subtypes of ADHD: A Retrospective Investigation
Source: Actas Esp Psiquiatr. 2026 Oct 15;54(5):1514–26. doi: 10.62641/aep.2224 (PMC13714461; doi:10.62641/aep.2224)
Supplement: Supplementary file 1 [file ActEsp-54-5-1514-1526-s1.zip › AEP-2224-layout-sup.docx]

**Association Among Systemic Immune-Inflammation Index, Vitamin D Status**

**and Clinical Subtypes of ADHD: A Retrospective Investigation**

Tingting Lu^1,^*, Yingying Chen^1^, Feiping Hu^1^, Yingping Zhu^2^

^1^Department of Child Health Care, Dongyang Maternal and Child Health Hospital, 322100 Jinhua, Zhejiang, China

^2^Healthcare Department, Dongyang Maternal and Child Health Hospital, 322100 Jinhua, Zhejiang, China

**Supplementary Materials**

**Supplementary Table 1. Primary clinical presentations and indications for outpatient evaluation in the non-ADHD comparison group (n = 40)**

| Primary clinical presentation | n (%) | Main reason for laboratory testing |
| --- | --- | --- |
| Attention-related concerns not meeting ADHD criteria | 20 (50.0) | Routine outpatient evaluation to exclude common organic contributors according to clinical judgment |
| Learning- or developmental-related concerns not meeting ADHD criteria | 15 (37.5) | Routine outpatient evaluation to assess developmental/learning concerns and exclude relevant organic conditions |
| Minor non-specific complaints | 5 (12.5) | Routine outpatient evaluation according to clinical judgment |
| Total | 40 (100) | Clinical outpatient evaluation |

Notes: ADHD, attention-deficit/hyperactivity disorder; DSM-5, Diagnostic and Statistical Manual of Mental Disorders, Fifth Edition. Data are presented as n (%). These children were included as a non-ADHD outpatient comparison group rather than as strictly healthy community controls. None met DSM-5 criteria for ADHD after clinical evaluation or the predefined exclusion criteria for acute infection, major neurodevelopmental disorders, major neurological disease, or severe chronic systemic illness.

**Supplementary Table 2. Comparison of clinically defined 25(OH)D categories between the ADHD and control groups stratified by season of blood draw**

| Season | Group | n | <20 ng/mL, n (%) | 20–29.9 ng/mL, n (%) | ≥30 ng/mL, n (%) | *p* |
| --- | --- | --- | --- | --- | --- | --- |
| Spring | Comparison | 9 | 0 (0.000) | 2 (22.22) | 7 (77.78) | 0.007 |
|  | ADHD | 13 | 1 (7.69) | 10 (76.92) | 2 (15.39) |  |
| Summer | Comparison | 10 | 1 (10.00) | 2 (20.00) | 7 (70.00) | 0.005 |
|  | ADHD | 24 | 2 (8.33) | 18 (75.00) | 4 (16.67) |  |
| Autumn | Comparison | 10 | 1 (10.00) | 3 (30.00) | 6 (60.00) | 0.048 |
|  | ADHD | 22 | 2 (9.09) | 16 (72.73) | 4 (18.18) |  |
| Winter | Comparison | 11 | 1 (9.09) | 2 (18.18) | 8 (72.73) | 0.002 |
|  | ADHD | 19 | 6 (31.58) | 11 (57.90) | 2 (10.53) |  |

Notes: ADHD, attention-deficit/hyperactivity disorder; 25(OH)D, 25-hydroxyvitamin D. Data are presented as n (%). Within each season, the distribution of clinically defined 25(OH)D categories (<20, 20–29.9, and ≥30 ng/mL) was compared between the ADHD and comparison groups using Fisher’s exact test. *p* values are two-sided.

**Supplementary Table 3. Sensitivity analysis restricted to in-house laboratory data only (n = 81)**

| Variable | Parsimonious model OR (95% CI) | *p* | Sensitivity model OR (95% CI) | *p* | Firth model OR (95% CI) | *p* |
| --- | --- | --- | --- | --- | --- | --- |
| Standardized SII | 8.14 (2.78–34.97) | <0.001 | 7.20 (2.12–37.34) | 0.007 | 3.80 (1.46-14.14) | <0.001 |
| Standardized 25(OH)D | 0.80 (0.32–1.72) | 0.585 | 0.62 (0.23–1.50) | 0.308 | 0.72 (0.32-1.47) | 0.373 |
| SII × 25(OH)D | 3.03 (1.46–7.53) | 0.007 | 3.80 (1.59–12.67) | 0.009 | 2.49 (1.26-6.48) | <0.001 |

Notes: ADHD, attention-deficit/hyperactivity disorder; BMI, body mass index; CI, confidence interval; CRP, C-reactive protein; OR, odds ratio; SII, systemic immune-inflammation index; 25(OH)D, 25-hydroxyvitamin D. The parsimonious model adjusted for age, sex, BMI, and season of blood draw. The sensitivity model additionally adjusted for parental education, residence type, and CRP. SII and 25(OH)D were entered as standardised z scores. The term SII × 25(OH)D denotes the interaction term between standardised SII and standardised 25(OH)D. *p* values are two-sided.
